# Elevation of erythrocyte sedimentation rate and C-reactive protein levels reflects renal interstitial inflammation in drug-induced acute tubulointerstitial nephritis

**DOI:** 10.1186/s12882-020-02175-z

**Published:** 2020-11-26

**Authors:** Xi-zi Zheng, Yang-hui Gu, Tao Su, Xu-jie Zhou, Jun-wen Huang, Ping-ping Sun, Yan Jia, Da-min Xu, Su-xia Wang, Gang Liu, Li Yang

**Affiliations:** 1 Renal Division, Peking University First Hospital, Peking University Institute of Nephrology; Key Laboratory of Renal Disease, Ministry of Health of China; Key Laboratory of Chronic Kidney Disease Prevention and Treatment (Peking University), Ministry of Education, Beijing, 100034 People’s Republic of China; 2 Renal Pathology Center, Peking University Institute of Nephrology, Beijing, 100034 People’s Republic of China; 3 Cardiovascular Division, Fourth Clinical Medical College, Guangzhou University of Chinese Medicine, Shenzhen, 518033 People’s Republic of China; 4 Laboratory of Electron Microscopy, Pathological Center, Peking University First Hospital, Beijing, 100034 People’s Republic of China

**Keywords:** C reactive protein, Erythrocyte sedimentation rate, Interstitial inflammation, Drug-induced acute tubulointerstitial nephritis

## Abstract

**Background:**

A renal biopsy is needed to define active inflammatory infiltration and guide therapeutic management in drug-induced acute tubulointerstitial nephritis (D-ATIN). However, factors such as various contraindications, refusal of informed consent and limited technical support may stop the biopsy process. It is thus of great importance to explore approaches that could deduce probable pathologic changes.

**Methods:**

A total of 81 biopsy-proven D-ATIN patients were enrolled from a prospective cohort of ATIN patients at Peking University First Hospital. The systemic inflammation score (SIS) was developed based on the CRP and ESR levels at biopsy, and patients were divided into high-SIS, median-SIS, and low-SIS groups. The demographic data, clinicopathologic features, and renal outcomes were compared.

**Results:**

The SIS was positively correlated with inflammatory cell infiltration and was inversely correlated with interstitial fibrosis. The number of interstitial inflammatory cells increased significantly with increasing SISs. The proportions of neutrophils and plasma cells were the highest in the high-SIS group compared with the other two groups. Prednisone (30–40 mg/day) was prescribed in all patients. The high-SIS group tended to have more favorable renal restoration than the other two groups. By 12 months postbiopsy, a decreased eGFR (< 60 mL/min/1.73 m^2^) was observed in 66.7% of medium-SIS patients, 32.4% of high-SIS patients, and 30.4% of low-SIS patients.

**Conclusion:**

The SIS was positively correlated with active tubulointerstitial inflammation and therefore could help to aid therapeutic decisions in D-ATIN.

## Background

Acute tubulointerstitial nephritis (ATIN) is a common renal lesion histopathologically characterized by inflammation and edema of the renal interstitium. It is responsible for 15–27% of acute kidney injury (AKI) [1–5], with drugs being the most common cause [6–10]. Unlike ischemic or toxic AKI, which usually induces acute tubular injury and results in an abrupt decline in renal function, patients with drug-induced ATIN (D-ATIN) sometimes have insidious renal dysfunction and are therefore more likely experience delayed recognition. A renal biopsy is needed to make a definitive diagnosis of D-ATIN and reveals the activity and severity of interstitial inflammation that usually directs immunosuppressive treatment [11–13]. However, factors such as various contraindications, refusal of informed consent and limited technical support may stop the process of renal biopsy. It is thus of great importance for those patients clinically suspected of having D-ATIN, in whom renal biopsy cannot be conducted, to explore approaches that could deduce probable pathologic changes and indicate the severity of interstitial inflammatory cell infiltration, which could therefore help make therapeutic decisions.

The erythrocyte sedimentation rate (ESR) and C-reactive protein (CRP) are traditional inflammatory markers that have been used to help assess the activity of inflammation in various diseases, such as systemic lupus erythematosus [14–17], rheumatoid arthritis [18, 19], and vasculitis [20]. However, there is still a lack of knowledge about the relevance of these systemic inflammatory parameters to renal inflammation. The current study was performed in a prospective cohort of D-ATIN patients. The clinical-pathological features and renal recovery of patients with different CRP and ESR levels were compared. Associations between systemic inflammation and renal tubulointerstitial inflammation as well as long-term renal outcomes were further analyzed.

## Methods

### Patients

The study was approved by the Committee on Research Ethics of Peking University First Hospital. Patients who were clinicopathologically diagnosed with D-ATIN from January 1, 2005, to December 31, 2018, and who were followed for at least 12 months were screened in a prospective cohort of ATIN patients in Peking University First Hospital as previously described [21]. The diagnosis of D-ATIN was made based on previously described criteria [22]. The presence of prominent interstitial inflammation in the nonfibrotic cortex and tubulitis was essential for the pathologic diagnosis of ATIN. All patients were screened for autoimmune diseases, malignancy and infectious diseases and accepted ophthalmological examinations to identify tubulointerstitial nephritis and uveitis syndrome (TINU) during their hospital stay. The cause of ATIN was reevaluated at every visit by the follow-up nephrologist group. Altogether, 81 patients with a final diagnosis of D-ATIN were enrolled in the current study.

### Clinical parameter evaluation and grouping for systemic inflammation

**Fig. 1:**
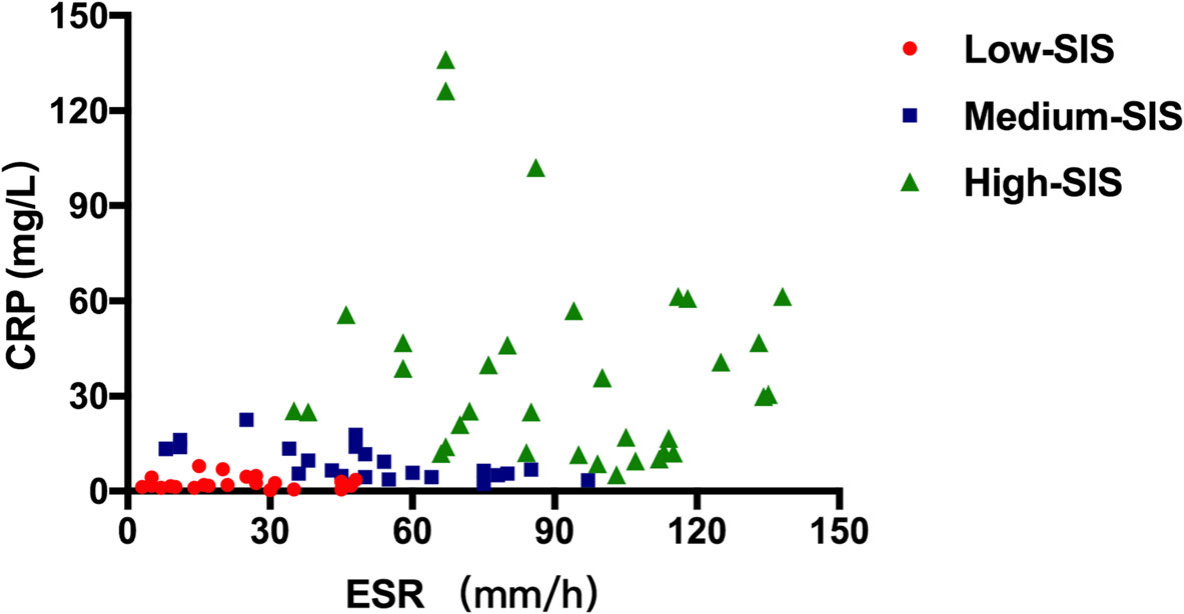
Scatter plot for CRP versus ESR values in different SIS groups

Clinical parameters and laboratory data were documented. Acute kidney disease (AKD) was defined using the Kidney Disease: Improving Global Outcomes (KDIGO) criteria [23] and consensus report of the Acute Disease Quality Initiative (ADQI) 16 Workgroup [24]. The ESR was assessed by the Westergren method (Greiner bio-one, Germany), and CRP was detected by rate nephelometry (IMMAGE 800, Beckman Coulter, America) at the time of biopsy. The ESR and CRP levels were categorized by quartile and ranked from 0 ~ 3 points (Table 1). The systemic inflammatory score (SIS) was calculated by combining the ESR and CRP points, and patients were classified into 3 systemic inflammation groups: low-SIS (score 0–1), medium-SIS (score 2–3) and high-SIS (score 4–6) groups. The scatter plot for CRP versus ESR values in different SIS groups was shown in Fig. 1.

**Table 1:** Scoring system based on values of ESR and CRP

CRP (mg/dL)	ESR (mm/hr)			
	< 30 (0 Point)	30–50 (1 Points)	50–86 (2 Points)	≥86 (3 Points)
< 3.6 (0 Point)	0	1	2	3
3.6–9.6 (1 Points)	1	2	3	4
9.6–25.0 (2 Points)	2	3	4	5
≥25.0 (3 Points)	3	4	5	6

### Evaluation of renal pathologic features

All kidney biopsy tissues were processed for light microscopy, immunofluorescence, and electron microscopy. Semiquantitative scores for interstitial edema, infiltration, fibrosis, tubulitis and tubular atrophy were assessed by two pathologists referring to a modification of the Banff Working Classification [25, 26]. The activity index was the total of the scores for interstitial edema, infiltration and tubulitis. The chronicity index was the total of the scores for interstitial fibrosis and tubular atrophy.

### Immunofluorescence staining of infiltrated inflammatory cells

Infiltrating cells were identified by immunofluorescence staining with antibodies against CD3 (T lymphocytes, 1:100, ZM-0417, ZSGB-BIO), CD20 (B lymphocytes, 1:100, ZA-0293, ZSGB-BIO), CD38 (plasma cells, 1:250, ab108403, Abcam), CD68 (monocytes/macrophages, 1:500, ZM-0060, ZSGB-BIO), and neutrophil elastic protease antibodies (neutrophils, 1:200, GWB-8F72C4, Genway-Bio) and were counted at 400x magnification. Eosinophils were detected with hematoxylin and eosin staining and counted at 200x magnification. The quantitation of tubulointerstitial infiltration was determined by averaging the counts of five randomly selected fields. The mean values are expressed as cells per millimeter squared.

### Follow-up, renal recovery and renal outcome

Serum creatine (sCr) was routinely performed during the follow-up periods. Renal recovery was based on sCr levels at 6 months postbiopsy. Complete recovery was defined as improvement in sCr levels to within 25% of baseline (or to 133 μmol/L if the baseline was not available); partial recovery as a > 50% decrease in sCr level from its peak value but not reaching within 25% of its baseline value; and no recovery as the failure to meet the criteria for complete or partial recovery or remaining on renal replacement therapy (RRT). The renal outcome was defined by the estimated glomerular filtration rate (eGFR) at 12 months postbiopsy. The eGFR was calculated by the Chronic Kidney Disease Epidemiology Collaboration (CKD-EPI) equation [27] and expressed as milliliters per minute per 1.73 m^2^.

### Statistical analysis

Analyses were performed using SPSS 22.0 Statistics software (IBM Corp., Armonk, NY) and GraphPad Prism version 6 (GraphPad Software, San Diego, CA). Categorical variables were expressed as counts and percentages. Continuous data with a normal distribution were presented as the mean and s.d., and those with an abnormal distribution were presented as the median and 25th–75th percentile. To assess group differences, one-way analyses of variance and Chi-squared analyses were conducted. Bonferroni post hoc comparisons were computed when significant differences emerged. Spearman’s rank correlation coefficient was used as a measure of correlations between the SIS and clinicopathological parameters. Multiple linear regression analysis was computed with the activity index as the dependent variable and with the following variables as explanatory variables: SIS, sex, age, disease course, and hemoglobin and eGFR levels at biopsy. Binary logistic regression analysis was used for predictors and ROC for cutoff points. A two-sided *P* < 0.05 was considered statistically significant.

## Results

### Baseline demographic and clinical data

As expressed in Table 2, the average age of the 81 D-ATIN patients was 45.4 ± 12.9 years, with a female predominance (51/81, 63.0%). The interval from the initiation of drug use to the diagnostic biopsy was 30 (14, 63) days. The majority of patients (77/81, 95.1%) were identified as having AKD, and 4 patients (4.9%) were classified with CKD. Eighteen patients (22.2%) required and initiated RRT before the biopsy. Seven patients (8.6%) were oliguric. Twenty patients (24.7%) had an allergic history. Common clinical features included digestive symptoms (61.7%), weakness (48.1%), fever (45.7%) and rash (16.0%). Beta-lactams, herbal medicine and nonsteroidal anti-inflammatory drugs were the most prevalent culprit agents (45.7, 39.5 and 32.1%, respectively). Thirty-eight patients (46.9%) were identified as using more than one kind of culprit drug.

**Table 2:** Demographic and clinical features in different SIS groups

Variables	Total *N* = 81	Low-SIS *N* = 23	Medium-SIS *N* = 24	High-SIS *N* = 34	*P*-value
Female, n(%)	51 (63.0)	11 (47.8)	16 (66.7)	24 (70.6)	0.197
Age (year)	45.4 ± 12.9	44.4 ± 13.2	47.5 ± 13.1	44.5 ± 12.96	0.643
Disease course (day)^a^	30 (14, 63)	30 (20, 52)	30 (20, 57)	17 (10, 60)	0.798
Allergic history, n(%)	20 (24.7)	6 (26.1)	6 (25.0)	8 (23.5)	0.975
Fever, n(%)	37 (45.7)	8 (34.8)	11 (45.8)	18 (52.9)	0.402
Rash, n(%)	13 (16.0)	4 (17.4)	3 (12.5)	6 (17.6)	0.847
AKD, n(%)	77 (95.1)	19 (82.6)	20 (100.0)*	27 (100.0)*	0.005
RRT, n(%)	18 (22.2)	1 (4.3)	9 (37.5)*	8 (23.5)*	0.023
Oliguria, n(%)	7 (8.6)	0 (0)	2 (8.3)	5 (14.7)	0.064
Suspected drug, n(%)					
Beta-lactams	37 (45.7)	11 (47.8)	8 (33.3)	18 (52.9)	0.326
Herbal medicine	32 (39.5)	13 (56.5)	10 (41.7)	9 (26.5)	0.072
NSAIDs	26 (32.1)	7 (30.4)	7 (29.2)	12 (35.3)	0.868
PPIs	6 (7.4)	2 (8.7)	4 (16.7)	0 (0.0)^#^	0.023
Laboratory tests					
SCr at peak (μmol/L)	322 (220, 531)	269 (158, 329)	417 (242, 646) *	358 (248, 521) *	0.021
SCr at biopsy (μmol/L)	249 (160, 386)	151 (118, 235)	274 (205, 585) *	321 (185, 449) *	< 0.001
Hematuria, n (%)	21 (25.9)	3 (13.0)	9 (37.5)	9 (26.5)	0.160
Leukocyturia, n (%)	46 (56.8)	8 (34.8)	11 (45.8)	27 (79.4)* ^#^	0.002
UTP (g/24 h)	1.1 (0.5, 1.5)	0.6 (0.2, 1.3)	1.1 (0.3, 1.5)	1.2 (0.9, 1.6) *	0.038
U-NAG (U/L)	26 (15, 47)	37 (16, 57)	20 (13, 33)	25 (15, 48)	0.215
U-α1MG (mg/L)	171 (74, 238)	76 (26, 218)	154 (73, 252)	206 (136, 271) *	0.031
U-mAlb (mg/L)	64 (36, 134)	49 (15, 134)	60 (36, 110)	75 (56, 159)	0.147
Renal glycosuria, n (%)	62 (76.5)	15 (65.2)	18 (75.0)	29 (85.3)	0.210
U-Osm decrease, n(%)	60 (74.1)	14 (60.9)	20 (83.3)	26 (76.5)	0.196
RTA, n (%)	43 (53.1)	6 (26.1)	15 (62.5) *	22 (64.7) *	0.009
Hemoglobin (g/L)	104.0 ± 16.6	114.7 ± 14.0	103.0 ± 20.2*	97.5 ± 12.6*	0.001
Hypokalemia, n (%)	37 (45.7)	9 (39.1)	10 (41.7)	18 (52.9)	0.853
ESR (mm/h)	61.0 ± 37.1	25.6 ± 14.3	48.8 ± 25.6*	91.5 ± 28.5*^#^	< 0.001
CRP (mg/L)	9.7 (3.6, 25.1)	1.9 (1.3, 3.6)	6.7 (4.9, 13.4) *	27.6 (12.3, 49.0)*^#^	< 0.001
IgG (g/L)	15.6 ± 4.4	14.0 ± 4.0	15.2 ± 3.3	16.9 ± 5.7	0.681
C3 (mg/L)	1.1 ± 0.3	0.9 ± 0.2	1.1 ± 0.2*	1.3 ± 0.2*^#^	< 0.001

Abbreviations: *SIS* systemic inflammatory score; *AKD* acute kidney disease; *RRT* renal replacement therapy; *NSAIDs* non-steroidal anti-inflammatory drugs; *PPIs* proton pump inhibitors; *sCr* serum creatinine; *eGFR* estimated glomerular filtration rate; *UTP* urinary total protein; *U-KIM1* urinary kidney injury molecular 1; *U-NAG* urinary N-acetyl-β-D-glucosaminidase; *U-α1MG* urinary α1 microglobulin; *U-mAlb* urinary microalbumin; *U-Osm* urinary osmolality; *RTA* renal tubular acidosis; *IgG* immunoglobulin G; *C3* complement 3

Normal range: U-NAG (0.3–12) U/L, U-α1MG (0–12) mg/L, U-mAlb (0–19) mg/L, ESR (0–15) mm/h, CRP (0–8) mg/L, IgG (7.2–16.9) g/L, C3 (0.6–1.5) mg/L

^a^ Disease course was defined as the interval from initiation of drug use to the diagnostic biopsy

^*^compared with low-SIS group, *P* < 0.05; ^#^ compared with medium-SIS group, *P* < 0.05

### Clinical relevance of the SIS in D-ATIN patients

Of the 81 D-ATIN patients, the ESR was elevated in 70 (86.4%), with an average level of 61.0 mm/hr. CRP was elevated in 44 patients (54.3%), with a median value of 9.7 mg/L (Table 2). SISs evaluated by both ESR and CRP levels were positively correlated with sCr values at renal biopsy (*r* = 0.440; *P* < 0.001), leukocyturia (*r* = 0.366; *P* = 0.001) and C3 levels (*r* = 0.533; *P* < 0.001) (Additional Table 1).

Based on the SIS values, there were 23 patients in the low-SIS group, 24 in the medium-SIS group and 34 in the high-SIS group. There was no significant difference in age, sex, allergic manifestations, or causal medications among the three groups of patients. Patients in the low-SIS group had the mildest kidney injuries, with the lowest sCr levels at renal biopsy (median value: 151 μmol/L, *P* < 0.001), lowest RRT rate (4.3%, *P* = 0.023) and highest hemoglobin concentration (114.7 ± 14.0 g/L, *P* = 0.001). It is interesting to note that patients in the medium-SIS group tended to have higher peak sCr levels (median 417 vs 358 μmol/L) and RRT rates (37.5% vs 23.5%) than those in the high-SIS group, yet their disease courses were relatively longer (median 30 vs 17 days) with lower levels of sCr at biopsy (median 274 vs 321 μmol/L). In addition, patients in the high-SIS group had significantly higher C3 levels (1.3 ± 0.2 vs 1.1 ± 0.2 mg/L, *P* < 0.001) with a greater prevalence of leukocyturia (79.4% vs 45.8%, *P* = 0.002) than those in the medium-SIS group (Table 2).

### Pathological relevance of the SIS in D-ATIN patients

Compared to patients in the low-SIS and medium-SIS groups, those in the high-SIS group had the highest degree of interstitial inflammation (*P* < 0.001) and the lowest degree of interstitial fibrosis (*P* = 0.030) (Table 3). The SIS was positively correlated with renal interstitial inflammatory cell infiltration (*r* = 0.508; *P* < 0.001) and interstitial edema (*r* = 0.294; *P* = 0.008) and inversely correlated with interstitial fibrosis (*r* = − 0.266; *P* = 0.016) (Additional Table 1). Multiple linear regression analysis demonstrated that only the SIS was significantly correlated with the renal activity index (β coefficient = 0.293, *P* = 0.003).

**Table 3:** Pathology features in different SIS groups

Variables	Total *N* = 81	Low-SIS *N* = 23	Medium-SIS *N* = 24	High-SIS *N* = 34	*P*-value
***Semiquantitative pathologic score***					
Activity index	4 (3, 5)	3 (2, 4)	3 (2, 4)	4 (4, 5)*^#^	< 0.001
Interstitial edema	1 (0, 1)	1 (0, 1)	1 (0, 1)	1 (1, 1)*	0.044
Interstitial inflammation	3 (2, 4)	2 (2, 3)	2 (2, 3)	4 (3, 4)*^#^	< 0.001
Tubulitis	0 (0, 0)	0 (0, 1)	0 (0, 1)	0 (0, 0)	0.218
Chronicity index	1 (0, 2)	1 (0, 3)	0 (0, 2)	1 (0, 2)	0.438
Interstitial fibrosis	0 (0, 1)	0 (0, 2)	0 (0, 2)	0 (0, 0)*	0.030
Tubular atrophy	0 (0, 2)	1 (0, 2)	0 (0, 1)	1 (0, 2)	0.629
***Interstitial inflammatory cell counts***					
Total cells^a^	391.5 ± 135.5	294.8 ± 145.4	378.2 ± 97.8	450.4 ± 124.0*	0.001
T lymphocytes	173.9 ± 63.6	137.6 ± 72.3	170.5 ± 64.0	195.5 ± 50.08*	0.019
B lymphocytes	45.4 (30.4, 62.0)	34.7 (14.4, 41.6)	45.6 (30.7, 60.4)	56.4 (40.1, 69.1)*	0.040
Monocytes/macrophages	101.8 ± 34.8	77.6 ± 35.9	90.7 ± 30.5	121.6 ± 24.9*^#^	< 0.001
Plasma cells	43.8 ± 24.3	30.3 ± 20.7	32.6 ± 19.3	57.9 ± 21.7*^#^	< 0.001
Neutrophils	15.4 (4.9, 33.0)	2.9 (1.7, 13.8)	7.9 (4.7, 20.4)	31.7 (15.7, 48.8)*^#^	< 0.001
Eosinophils^b^	2.1 (0.5, 5.1)	0.4 (0.1, 1.7)	0.8 (0.0, 1.9)	3.8 (2.2, 9.0) *^#^	< 0.001
***Percentages of Interstitial inflammatory cells (%)***					
T lymphocytes	44.3 ± 9.0	46.9 ± 8.5	46.1 ± 10.1	41.7 ± 8.2	0.136
B lymphocytes	11.7 (9.1, 14.2)	10.6 (8.8, 14.5)	12.6 (9.4, 14.9)	11.5 (8.7, 14.0)	0.263
Monocytes/macrophages	26.0 (21.3, 29.9)	25.8 (21.5, 30.9)	25.7 (19.9, 30.1)	26.2 (23.4, 29.1)	0.670
Plasma cells	10.9 ± 5.2	9.4 ± 4.4	9.2 ± 5.7	12.6 ± 4.9*^#^	0.047
Neutrophils	4.2 (1.6, 7.8)	1.1 (0.9, 5.4)	2.4 (1.7, 4.8)	7.5 (4.3, 11.7)*^#^	< 0.001

Abbreviations: *SIS* systemic inflammatory score

^a^ Total cells count was the sum of T lymphocytes, B lymphocytes, macrophages, plasma cells and neutrophil under 400× magnification

^b^ Eosinophils were counted under 200× magnification

*: compared with low-SIS group, *P* < 0.05; ^#^: compared with medium-SIS group, *P* < 0.05

We next investigated renal interstitial inflammatory cell types through immunofluorescence staining. The number of each kind of interstitial inflammatory cell increased significantly with the increase in SISs (Table 3). When focusing on the constitution of inflammatory cells, the proportions of neutrophils (7.5% vs 2.4% in medium-SIS vs 1.1% in low-SIS; *P* < 0.001) and plasma cells (12.6% vs 9.2% in medium-SIS vs 9.4% in low-SIS; *P* = 0.047) were the highest in patients in the high-SIS group compared with those in the other two groups. There was no significant difference in the proportions of T lymphocytes, B lymphocytes or macrophages among the three groups of patients. Eosinophils, which favor a diagnosis of drug-induced ATIN, were also highest in the high-SIS group (median value: 3.8 vs 0.8 in medium-SIS vs 0.4 in low-SIS; *P* < 0.001).

### Treatment and outcome among three groups with different SISs

As shown in Table 4, prednisone was prescribed at a dosage of 30–40 mg/day in all the patients. Additional immunosuppressive agents, such as mycophenolate, azathioprine and cyclophosphamide, were used in 27.2% (22/81) of patients, with no significant difference among the three groups (*P* = 0.436). Methylprednisolone pulse therapy was performed in 22.2% (18/81) of all patients, and none of the low-SIS patients received methylprednisolone pulse therapy.

**Table 4:** Treatment and renal outcome in different SIS groups

Variables	Total *N* = 81	Low-SIS *N* = 23	Medium-SIS *N* = 24	High-SIS *N* = 34	*P*-value
Immunosuppressive treatment, n (%)					
Prednisone only	52 (64.2)	19 (82.6)	15 (62.5)	18 (52.9)	0.071
Methylprednisolone pulse therapy	18 (22.2)	0 (0)	6 (25.0)*	12 (35.3)*	0.007
Immunosuppressive medications	22 (27.2)	4 (17.4)	8 (33.3)	10 (29.4)	0.436
Renal recovery at 6 months post-biopsy, n(%)					
Complete	52 (64.2)	15 (65.2)	12 (50.0)	25 (73.5)	0.195
Partial	28 (34.6)	7 (30.4)	12 (50.0)	9 (26.5)	
None	1 (1.2)	1 (4.3)	0 (0)	0 (0)	
Renal outcome evaluated by eGFR (mL/min/1.73 m^2^)					
≥ 60	47 (58.0)	16 (69.6)	8 (33.3)*	18 (67.6)^#^	0.014
< 60	28 (42.0)	7 (30.4)	16 (66.7)	11 (32.4)	

Abbreviations: *SIS* systemic inflammatory score; *eGFR* estimated glomerular filtration rates; *CKD* chronic kidney disease; *CRP* C reactive protein; *ESR* erythrocyte sedimentation rate

*: compared with low-SIS group, *P* < 0.05; ^#^: compared with medium-SIS group, *P* < 0.05

**Fig. 2:**
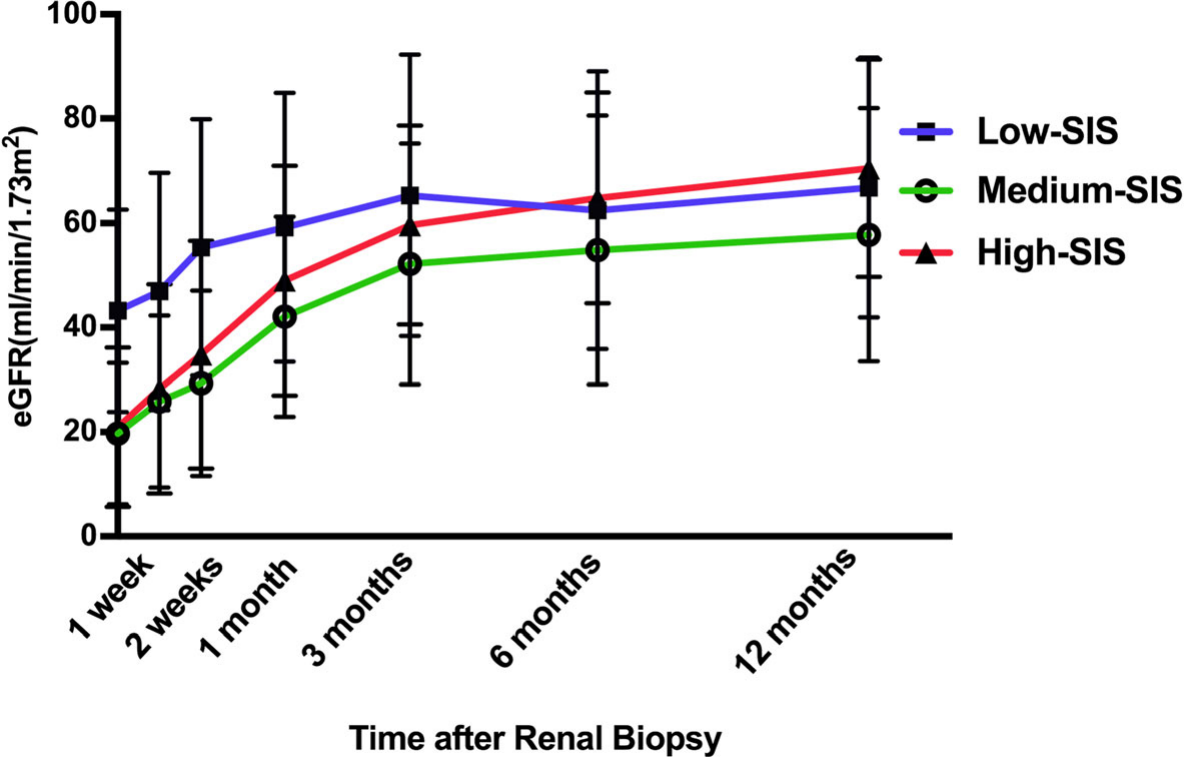
The restoration of renal function in different SIS groups during the first year postrenal biopsy. Patients in both the medium-SIS and high-SIS groups had significant renal dysfunction at the time of renal biopsy, and high-SIS patients presented with more rapid renal function restoration. Low-SIS patients had modest renal dysfunction and modest restoration of renal function

Patients were followed for at least 12 months (range: 12–132 months, median 38 months). The high-SIS group tended to have more favorable renal restoration than the other two groups (Fig. 2). At 6 months postbiopsy, complete recovery was achieved in 73.5% of high-SIS patients, 50.0% of medium-SIS patients, and 65.2% of low-SIS patients (*P* = 0.195). A decreased eGFR (< 60 mL/min/1.73 m^2^) was observed in 32.4% of high-SIS patients, 66.7% of medium-SIS patients, and 30.4% of low-SIS patients (*P* = 0.014) at 12 months postbiopsy (Table 4). Adding SIS as a continuous variable to age and eGFR measured at biopsy made a small increase for the area under receiver operating characteristic curve by using the logistic regression analysis (from 0.696 to 0.731 for complete recovery at 6 months and from 0.852 to 0.875 for decreased eGFR at 12 months).

Fifty-four patients with severe renal dysfunction at the time of biopsy (eGFR < 30 mL/min/1.73 m^2^) were divided into high-score (*N* = 26) and low-score (*N* = 28) subgroups based on the SIS. The eGFR values at biopsy were similar in the two subgroups (14.3 ± 7.8 mL/min/1.73 m^2^ in high-score vs 17.7 ± 8.5 in low-score, *P* = 0.131). At 12 months postbiopsy, the eGFR values were significantly higher in the high-score subgroup (65.3 ± 20.2 vs 52.9 ± 20.9 mL/min/1.73 m^2^ in low-score, *P* = 0.032).

## Discussion

D-ATIN is a relatively common cause of AKI. Previously, the ESR and CRP were reported to be significantly elevated in D-ATIN patients [13, 28]. The current study first demonstrated that the SIS evaluated by the ESR and CRP was correlated with active renal tubulointerstitial inflammation and renal restoration in a prospective cohort of D-ATIN patients and therefore could help to aid therapeutic decisions when a renal biopsy is not acceptable or cannot be performed serially in this disease condition.

Patients with D-ATIN often present with a relatively insidious onset and a disease process of subacute renal dysfunction, with oliguria not commonly seen [13]. Therefore, a delayed diagnosis is likely to be encountered, especially when patients initially present at non-nephrology departments, as shown in our study, where the median time course from the initial use of suspicious drugs to diagnostic renal biopsy was 30 days, even exceeding 3 months in some cases. Once acute interstitial inflammation sets in, it can progress rapidly to a less reversible, more destructive fibrogenic process [29]. Therefore, the delay in diagnosis and treatment may result in complexity in the pathophysiologic process containing both active inflammation and fibrotic lesions in patients with D-ATIN. It is crucial to make a treatment decision with evidence that reflects renal interstitial active inflammation, yet renal biopsy, the gold standard for histological evaluation, is invasive and not acceptable for all patients. Following multivariate analysis, we observed that systemic inflammatory markers were closely correlated with renal inflammation regardless of the disease course. More importantly, it has been reported that D-ATIN patients might experience recurrent kidney injury during long-term follow-up due to various medications [21]. Elevated systemic inflammatory markers combined with abnormal urinary markers during follow-up may play an important role in reflecting the degree of renal inflammation and providing proper management for these patients.

During follow-up, for patients with similar eGFR values at biopsy, better renal outcomes were achieved in those with higher systemic inflammatory scores, suggesting that the SIS might serve as an indicator of renal outcome. Regarding pathological findings, the SIS was positively correlated with renal interstitial inflammatory cell infiltration, especially with neutrophils and eosinophils, which participate in the acute phase of inflammation [30], and was inversely correlated with interstitial fibrosis, which suggests its ability to reflect the activity of renal interstitial inflammatory injury in D-ATIN. A positive correlation was also observed between the SIS and plasma cell ratio. Plasma cell infiltration was found to be positively correlated with CRP levels and tubulointerstitial inflammation scores in antineutrophil cytoplasmic autoantibody-associated vasculitis [31]. The radical role of plasma cells in D-ATIN remains unclear, but we suppose that aggregated plasma cells in the kidney may play a role in local inflammation in the early phase. In summary, our findings indicate that the SIS may serve as a noninvasive biomarker for ongoing inflammatory processes and active kidney injury in D-ATIN patients, who might benefit from prompt treatment.

Our study has limitations related to the retrospective observational design. Due to the limited sample size of the patients that had both renal biopsy-proven D-ATIN and scheduled follow-up for at least 1 year, we were not able to test the scoring system in a new set of patients. Further study in a larger independent D-ATIN population is needed to validate our findings. CRP and ESR values are nonspecific markers that could be elevated in various conditions, such as infection and systemic autoimmune diseases. Therefore, the introduction of SISs in D-ATIN should be implemented after excluding these conditions.

## Conclusions

Our study first demonstrated the relationship between systemic inflammation and local renal inflammation in D-ATIN. The SIS based on ESR and CRP values may serve as a marker of the activity of tubulointerstitial inflammation and assist with decision-making in the immunosuppressive treatment of D-ATIN.

## Supplementary Information

**Additional file 1: Table S1**. Correlation of systemic inflammatory markers with clinicopathological parameters.

## Data Availability

The datasets used and analyzed during the current study are available from the corresponding author upon reasonable request.

## Abbreviations

D-ATIN: Drug-induced acute tubulointerstitial nephritis
CRP: C reactive protein
ESR: Erythrocyte sedimentation rate
SIS: Systemic inflammation score
HPF: High power field
sCr: Serum creatine
AKD: Acute kidney disease
KDIGO: Kidney Disease: Improving Global Outcomes
ADQI: The Acute Disease Quality Initiative
RRT: Renal replacement therapy
eGFR: Estimated glomerular filtration rate
CKD-EPI: Chronic Kidney Disease Epidemiology Collaboration
CKD: Chronic kidney disease
NSAIDs: Non-steroidal anti-inflammatory drugs
PPIs: Proton pump inhibitors
UTP: Urinary total protein
U-KIM1: Urinary kidney injury molecular 1
U-NAG: Urinary N-acetyl-β-D-glucosaminidase
U-α1MG: Urinary α1 microglobulin
U-mAlb: Urinary microalbumin
U-Osm: Urinary osmolality
RTA: Renal tubular acidosis
IgG: Immunoglobulin G
C3: Complement 3
