# Supplementary material for: Elevation of erythrocyte sedimentation rate and C-reactive protein levels reflects renal interstitial inflammation in drug-induced acute tubulointerstitial nephritis
Source: BMC Nephrol. 2020 Nov 26;21:514. doi: 10.1186/s12882-020-02175-z (PMC7689990; doi:10.1186/s12882-020-02175-z)
Supplement: Supplementary file 1 — Additional file 1: Table S1. Correlation of systemic inflammatory markers with clinicopathological parameters. [file 12882_2020_2175_MOESM1_ESM.docx]

**Additional Table 1. Correlation of systemic inflammatory markers with clinicopathological parameters**

|  | CRP | | ESR | | SIS | |
| --- | --- | --- | --- | --- | --- | --- |
|  | CC | *P* | CC | *P* | CC | *P* |
| ***Laboratory tests*** |  |  |  |  |  |  |
| SCr at peak (μmol/L) | 0.163 | 0.146 | 0.156 | 0.170 | 0.222 | 0.046 |
| SCr at biopsy (μmol/L) | 0.338 | 0.002 | 0.364 | 0.001 | 0.440 | <0.001 |
| Hematuria, n (%) | 0.124 | 0.270 | -0.088 | 0.441 | 0.054 | 0.635 |
| Leukocyturia, n (%) | 0.283 | 0.010 | 0.399 | <0.001 | 0.366 | 0.001 |
| U-NAG (U/L) | -0.085 | 0.469 | 0.020 | 0.866 | -0.099 | 0.403 |
| U-α1MG (mg/L) | 0.209 | 0.080 | 0.336 | 0.004 | 0.303 | 0.010 |
| U-mAlb (mg/L) | 0.184 | 0.116 | 0.276 | 0.018 | 0.210 | 0.072 |
| Renal glycosuria, n (%) | 0.130 | 0.249 | 0.319 | 0.004 | 0.258 | 0.020 |
| U-Osm<500mOsm/kg.H_2_O, n(%) | 0.126 | 0.256 | 0.038 | 0.738 | 0.133 | 0.235 |
| RTA, n (%) | 0.231 | 0.038 | 0.240 | 0.033 | 0.286 | 0.010 |
| Hemoglobin (g/L) | -0.284 | 0.010 | -0.554 | <0.001 | -0.507 | <0.001 |
| IgG (g/L) | -0.138 | 0.227 | 0.347 | 0.002 | 0.245 | 0.031 |
| C3 (mg/L) | 0.514 | <0.001 | 0.473 | <0.001 | 0.533 | <0.001 |
| ***Semiquantitative pathologic score*** | | | | | | |
| Activity index | 0.477 | <0.001 | 0.504 | <0.001 | 0.547 | <0.001 |
| Interstitial edema | 0.225 | 0.044 | 0.344 | 0.002 | 0.294 | 0.008 |
| Inflammatory cell infiltration | 0.452 | <0.001 | 0.458 | <0.001 | 0.508 | <0.001 |
| Tubulitis | 0.065 | 0.562 | 0.002 | 0.987 | 0.061 | 0.586 |
| Chronicity index | -0.035 | 0.755 | -0.205 | 0.070 | -0.082 | 0.469 |
| Interstitial fibrosis | -0.190 | 0.089 | -0.401 | <0.001 | -0.266 | 0.016 |
| Tubular atrophy | 0.101 | 0.369 | -0.007 | 0.954 | 0.078 | 0.488 |
| ***Interstitial inflammatory cell counts*** | | | | | | |
| Total cells^a^ | 0.458 | <0.001 | 0.204 | 0.128 | 0.415 | <0.001 |
| T lymphocytes | 0.276 | 0.039 | 0.166 | 0.229 | 0.275 | 0.041 |
| B lymphocytes | 0.385 | 0.003 | 0.244 | 0.075 | 0.359 | 0.007 |
| Monocytes/macrophages | 0.530 | <0.001 | 0.372 | 0.006 | 0.534 | <0.001 |
| Plasma cells | 0.359 | 0.007 | 0.349 | 0.010 | 0.473 | <0.001 |
| Neutrophils | 0.531 | <0.001 | 0.453 | 0.001 | 0.594 | <0.001 |
| Eosinophils^b^ | 0.510 | <0.001 | 0.419 | 0.003 | 0.565 | <0.001 |
| ***Percentages of Interstitial inflammatory cells (%)*** | | | | | | |
| T lymphocytes | -0.292 | 0.029 | -0.283 | 0.038 | -0.300 | 0.025 |
| B lymphocytes | 0.039 | 0.777 | -0.019 | 0.893 | 0.005 | 0.971 |
| Monocytes/macrophages | 0.060 | 0.661 | 0.116 | 0.402 | 0.070 | 0.608 |
| Plasma cells | 0.153 | 0.260 | 0.261 | 0.057 | 0.314 | 0.018 |
| Neutrophils | 0.438 | 0.001 | 0.414 | 0.003 | 0.493 | <0.001 |

Abbreviations: CRP, C reactive protein; ESR, erythrocyte sedimentation rate; RTA, renal tubular acidosis; SIS, systemic inflammatory score; CC, Correlation Coefficient;

^a^ Total cells count was the sum of T lymphocytes，B lymphocytes，macrophages，plasma cells and neutrophil under 400× magnification. ^b^ Eosinophils were counted under 200× magnification.
